# Detecting extra-ocular *Chlamydia trachomatis* in a trachoma-endemic community in Ethiopia: Identifying potential routes of transmission

**DOI:** 10.1371/journal.pntd.0008120

**Published:** 2020-03-04

**Authors:** Anna Last, Bart Versteeg, Oumer Shafi Abdurahman, Ailie Robinson, Gebeyehu Dumessa, Muluadam Abraham Aga, Gemechu Shumi Bejiga, Nebiyu Negussu, Katie Greenland, Alexandra Czerniewska, Nicholas Thomson, Sandy Cairncross, Virginia Sarah, David Macleod, Anthony W. Solomon, James Logan, Matthew J. Burton

**Affiliations:** 1 Clinical Research Department, London School of Hygiene & Tropical Medicine, London, United Kingdom; 2 The Fred Hollows Foundation, Ethiopia; 3 Department of Disease Control, London School of Hygiene & Tropical Medicine, London, United Kingdom; 4 Oromia Regional Health Bureau, Addis Ababa, Ethiopia; 5 Federal Ministry of Health, Addis Ababa, Ethiopia; 6 Department of Pathogen Molecular Biology, London School of Hygiene & Tropical Medicine, London, United Kingdom; 7 Parasites and microbes, Wellcome Trust Sanger Institute, Wellcome Trust Genome Campus, Hinxton, United Kingdom; 8 The Fred Hollows Foundation, London, United Kingdom; 9 Department of Infectious Disease Epidemiology, London School of Hygiene & Tropical Medicine, London, United Kingdom; RTI International, UNITED REPUBLIC OF TANZANIA

## Abstract

**Background:**

Trachoma elimination efforts are hampered by limited understanding of *Chlamydia trachomatis (Ct)* transmission routes. Here we aimed to detect *Ct* DNA at non-ocular sites and on eye-seeking flies.

**Methods:**

A population-based household survey was conducted in Oromia Region, Ethiopia. Ocular and non-ocular (faces, hands, clothing, water containers and sleeping surfaces) swabs were collected from all individuals. Flies were caught from faces of children. Flies, ocular swabs and non-ocular swabs were tested for *Ct* by quantitative PCR.

**Results:**

In total, 1220 individuals in 247 households were assessed. Active trachoma (trachomatous inflammation—follicular) and ocular *Ct* were detected in 10% and 2% of all-ages, and 21% and 3% of 1–9-year-olds, respectively. *Ct* was detected in 12% (95% CI:8–15%) of tested non-ocular swabs from ocular-positive households, but in none of the non-ocular swabs from ocular-negative households. *Ct* was detected on 24% (95% CI:18–32%) of flies from ocular-positive households and 3% (95% CI:1–6%) of flies from ocular-negative households.

**Conclusion:**

*Ct* DNA was detected on hands, faces and clothing of individuals living in ocular-positive households suggesting that this might be a route of transmission within *Ct* infected households. In addition, we detected *Ct* on flies from ocular-positive households and occasionally in ocular-negative households suggesting that flies might be a vector for transmission within and between *Ct* infected and uninfected households. These potential transmission routes may need to be simultaneously addressed to suppress transmission.

## Author summary

Trachoma elimination efforts are hampered by limited understanding of *Ct* transmission routes. One previous study demonstrated *Ct* in nasal secretions, with lower loads than in paired ocular samples. Two other studies reported 15–23% of *Musca sorbens* caught leaving the faces of Ethiopian children to be PCR-positive for *Ct* in untreated villages with 30–50% TF prevalence. To date, no published studies have systematically documented the relative frequency of *Ct* by PCR on non-ocular surfaces and flies. Cross-sectional studies consistently find active trachoma and ocular *Ct* infection associated with factors such as dirty faces, fly-eye contact, limited water access, crowded living conditions and limited sanitation. In this study, we conducted a population-based household survey in Oromia Region, Ethiopia and test the hypothesis that *Ct* can be detected at multiple non-ocular sites and on eye-seeking flies. The results of our study show that *Ct* DNA was only detected on hands, faces and clothing of individuals living in ocular-positive households. In addition, *Ct*-positive flies were much more likely to be found in ocular-positive households. These findings suggest there may be several plausible ocular *Ct* transmission routes between people, within and between households, which would need to be simultaneously addressed within communities to suppress transmission.

## Introduction

Trachoma, a neglected tropical disease, is the most common cause of infectious blindness globally, affecting some of the world’s poorest people [1]. Trachoma is caused by repeated ocular infection with the bacterium *Chlamydia trachomatis* (*Ct*). In trachoma-endemic populations, infection is most common in children and is associated with signs of active (inflammatory) trachoma. A key active trachoma sign is “trachomatous inflammation—follicular” (TF), the prevalence of which in 1–9-year-olds is used to guide decisions on district-level intervention. Chronic inflammation results in immunologically mediated conjunctival scarring and may lead to in-turned eyelashes scratching the eye, a manifestation of trachoma known as trachomatous trichiasis (TT). Eventually, in some individuals, sight is lost from irreversible corneal opacification [1].

The latest global estimates indicates that 158 million people live in trachoma-endemic areas and 2.8 million have TT [2, 3]. Around 1.9 million are blind or visually impaired. [4, 5]. More than 80% of the burden of active trachoma is concentrated in 14 countries in Sub-Saharan Africa, with Ethiopia being the country bearing the greatest burden [2].

Trachoma elimination efforts are hampered by limited understanding of *Ct* transmission routes and their relative importance. Transmission of ocular *Ct* from infected to uninfected individuals is hypothesised to occur directly through close contact or indirectly on eye-seeking flies and fomites (e.g. face cloths, towels, items of clothing) [1, 6–9]. However, detailed studies on transmission are lacking. We have previously demonstrated *Ct* in nasal secretions, with lower loads than in paired ocular samples [10]. Others have reported 15–23% of *Musca sorbens* caught leaving the faces of Ethiopian children to be PCR-positive for *Ct* in untreated villages with 30–50% TF prevalence [11, 12]. To date, no published studies have systematically documented the relative frequency of *Ct* by PCR on non-ocular surfaces and flies. This is a logical first step in developing rational approaches to suppressing transmission. Cross-sectional studies consistently find active trachoma and ocular *Ct* infection associated with factors such as dirty faces, fly-eye contact, limited water access, crowded living conditions and limited sanitation [13–18]. Though it is biologically plausible that these and other factors contribute to *Ct* transmission, finding an association is not a demonstration of causality. A better understanding of transmission requires longitudinal study using *Ct* PCR to identify potential transmission routes.

Here we test the hypothesis that *Ct* can be detected at multiple non-ocular sites and on eye-seeking flies, providing evidence for multiple potential transmission routes. Furthermore, by systematically mapping *Ct* detection at specific locations in trachoma endemic households, we aimed to infer an indication of their relative importance to transmission in this setting.

## Methods

### Study location

This study was conducted in Shashemane *woreda* (district), Oromia Region, Ethiopia. The most recent Global Trachoma Mapping Project (GTMP) data estimated an overall TF prevalence in 1-9-year-olds of 45.8% [19]. In Oromia, azithromycin Mass Drug Administration (MDA) is conducted using a community-based model whereby health extension workers distribute medications at a central point in each village. We undertook fieldwork between January and June 2018. One previous round of MDA had been conducted in this *woreda* in July 2016. The *woreda* was retreated in December 2018. Reported treatment coverage estimates for this district were over 80%.

### Pilot study

A pilot study was conducted in nine households. These were purposively selected following ocular examination of a convenience sample of children aged 1–9 years from an area of Shashemane *woreda* with high TF prevalence. All consenting individuals from the respective households were screened for active trachoma (TF and/or trachomatous inflammation—intense; TI) using the WHO simplified grading system [20] by a qualified trachoma grader. The pilot study was conducted to inform decisions about testing and analysis of samples collected in the main population-based survey.

### Population-based survey

Following the pilot study, we randomly selected households within a geographically contiguous area of Shashemane *woreda*. For inclusion in the population-based survey, a household was required to have at least one child aged 1–9 years resident on the day of enumeration. All members of the household were eligible to participate. The 247 selected households were enumerated, and basic socio-demographic data were collected, including age, gender. GPS coordinates of households were recorded.

### Clinical assessment

In both the pilot and the population-based household survey, all individuals (aged six months and above) were examined for clinical signs of trachoma. In brief, a qualified trachoma grader examined the upper tarsal conjunctiva of both eyes using a 2.5× binocular loupe and graded using the simplified Trachoma Grading System [20]. Faces were assessed for the presence of ocular and/or nasal discharge and the presence of flies on the face. Ophthalmic nurses were trained and instructed to make an assessment of facial cleanliness by examining the face for ocular and nasal discharge and the presence of flies on the face for 30 seconds. In order to assign the presence of ocular discharge, active discharge from the eye must have been present. Simple eyelash crusting was not sufficient to assign the presence of ocular discharge. To assign the presence of nasal discharge, there must have been active discharge from one or both nostrils. Simple crusting around the nose was not sufficient to assign the presence of nasal discharge. Hands were assessed for the presence of visible dirt and/or secretions.

### Ocular swab collection

After clinical assessment, an ophthalmic nurse wearing examination gloves collected a conjunctival swab sample from the left upper tarsal conjunctiva. A sterile dacron swab (Puritan) was wiped four times across the everted tarsal conjunctival surface, rotating the shaft by 90° with each sweep. Swabs were stored in 500 μL of 0.2 M-sucrose-phosphate (2SP) transport medium. Gloves were changed between participants. Air control swabs were collected by holding a swab in the air in front of the participant’s eye for 20 seconds and treated in the same way as clinical samples. One air control swab was randomly collected for each 50 sample swabs in the field to evaluate field and laboratory contamination. All samples were placed immediately in a cool box with ice blocks in the field and transferred to a -20°C freezer at the end of each day. The swabs were subsequently stored at -80°C in the Oromia Regional Health Laboratory, Adama, Oromia, until testing.

### Non-ocular swab collection

Non-ocular swabs were systematically collected from faces and from the finger pads and dorsum of each hand of all consenting individuals in a household. Swabs were also taken from five potential fomite sites: (1) cuff and (2) neckline of clothing, (3) large plastic water collection cans, (4) washing jugs and (5) sleeping surfaces. Swabs for use at non-ocular sites were pre-moistened in 2SP and systematically rubbed with moderate and consistent pressure across an identified surface of 10cm^2^, horizontally and vertically for ten seconds. Non-ocular swabs handled and processed in the same way as the ocular swabs. One air control fomite swab was randomly collected for each 50 sample swabs using methods described above.

### Flies

In each household we attempted to catch flies from the faces of two children, preferentially aged 1–9 years, who were chosen by the visiting field worker. Flies landing on the child’s face were captured by disturbing and catching with an insect net (Bugdorm 38cm diameter). The nets were sterilised after use by immersion in freshly prepared 1:100 Virkon-S for ten minutes, followed by rinsing in bottled water, and air drying in the sun. Catches were attempted for 15 minutes or until 10 flies were caught, whichever came first. Each captured fly was immediately transferred from the net to an individual ziplock bag that had the bottom corners cut off. All bagged flies caught from a single child were then placed into another (complete) bag containing a ball of cotton wool soaked in 50% acetone-alcohol and placed in a cool box. Immediate killing with acetone, followed by cooling, served to minimise fly grooming behaviours prior to death. Flies were taken to our local laboratory in Shashemane, where they were transferred individually into sterile tubes using forceps that had been sterilised using Virkon-S. Samples were temporarily stored at -20°C before transfer to the -80°C freezer in Adama, where they were stored until testing. Five flies per child were separately tested by *Ct* qPCR; where more than five flies had been captured, the first five that were stored for each child were chosen.

### DNA extraction

DNA was extracted using the Biochain Blood and Serum kit (AMS Biotechnology Europe Ltd). Swabs were vortexed in the 500μL of 2SP at full speed for two minutes. The swab was removed and discarded, expressing any excess liquid on the side of the tube. Flies were pre-treated with 200μL digestion buffer containing 98% dH_2_O, 1% 1M Tris pH 8.0 (VWR International), 1% Proteinase K 20mg/mL (Life Technologies), 0.5% 5M NaCl (Invitrogen) and 0.2% 0.5M EDTA (VWR international). After adding the digestion buffer, flies were incubated for 30 minutes at 37°C and two minutes at 95°C. DNA extraction of all samples was then completed following the manufacturer's recommendations and eluted in 80μL TE-buffer. The eluate was stored at -80°C and thawed once for *Ct* detection.

### *Chlamydia trachomatis* quantification and load estimation

*Ct* detection was performed using an in-house multiplex quantitative PCR (qPCR) assay targeting the *Ct* chromosomal *omcB* gene, plasmid *pORF2* gene and human *RPP30* gene (the last of which functioned as an internal control to confirm adequacy of sample collection, extraction and PCR), as previously described [21]. The assay was performed on a Quantstudio 7 flex Real-Time PCR machine (Applied Biosystems) in 384-well format. Additionally, non-ocular and fly eluates were spiked with human DNA obtained from a HEP2 cervical cell line prior to testing, to detect possible inhibition.

QuantStudio software v1.3 (Applied Biosystems) was used for PCR-data analysis. Samples were classified as *Ct-*positive if amplification of the *omcB* or *pORF2* target was detected within 40 cycles. *Ct* load was estimated by extrapolation from an eight-step, ten-fold dilution of standards of known concentration; these were tested in duplicate on each plate.

### Statistical analysis and geographical mapping

A person was considered ocular-positive if they had a positive ocular swab. Individual ocular *Ct* load categories were defined as high- or low-load based on the median ocular *omcB* load (since *Ct* are known to have only one chromosome copy of *omcB*, but variable plasmid *pORF2* copy numbers) [22]. Household *Ct* load categories were defined as high- or low-load based on the highest individual ocular *Ct* load category found in each household. Households were considered positive if at least one resident was ocular-positive.

Differences in characteristics between ocular-positive households and a random selection of ocular-negative households were tested using the Pearson X^2^ test for categorical data. Fisher’s exact test was used when an expected cell count was <1. For age, as a continuous variable, the Mann-Whitney U test was used. All analyses were performed using R version 3.4.2 (The R Foundation for Statistical Computing, 2017).

For geographical mapping, household level qPCR data from ocular swabs, non-ocular swabs and flies were linked to household GPS coordinates and projected onto OpenStreetMap using ArcGIS 10.5 (ESRI Inc., USA). Geospatial cluster analysis of *Ct*-positive households was performed using the k-means method, which groups observations into k-clusters based on geographical distance between household GPS coordinates. The optimal number of clusters was defined by calculating the within sum of squares for a number of clusters using a scree plot.

### Ethics

This study was conducted in accordance with the declaration of Helsinki. Ethical approval was given by the Ethics Committees of the London School of Hygiene & Tropical Medicine, Ethiopian Federal Ministry of Science and Technology and Oromia Regional Health Bureau. Verbal consent was obtained from community leaders. Written informed consent was provided by all participants or (for children) their guardians.

## Results

### Pilot study

Fifty-seven individuals from nine households in two villages were enrolled in the pilot study between 16^th^ and 20^th^ January 2018. The median age of participants was 7 years (range 1–55 years); 25/57 (44%) were female (Table 1). No ocular discharge was reported, but around half (30/57; 53%) had flies on their faces at examination, and 20/57 (3%) had nasal discharge (Table 1).

**Table 1:** Characteristics 57 individuals living in the nine pilot households, by ocular *C*. *trachomatis* qPCR status.

Variable		Total (n = 57)		*C*. *trachomatis* positive (n = 11)^a^
	n	%	n	%
Age in years continuous				
Median (IQR)	7	(6–28)	6	(6–28)
Age years categorical				
1–9	33	57.9	8	72.7
10–17	9	15.8	1	9.1
18+	15	26.3	2	18.2
Sex				
Female	25	43.9	5	45.5
Male	32	56.1	6	54.5
Presence of ocular discharge				
Yes	0	0.0	0	0.0
Presence of nasal discharge				
Yes	20	35.1	6	54.5
Presence of flies on the face				
Yes	30	52.6	8	72.7
Presence of TF				
Yes	23	40.4	8	72.7
Presence of TI				
Yes	7	12.3	7	63.6
Presence of TF and/or TI				
Yes	23	40.4	8	72.7

Abbreviations: TF, trachomatous inflammation—follicular; TI trachomatous inflammation—intense; IQR, Inter quartile range.

^a^Shows column percentage compared to all *Chlamydia trachomatis* qPCR positive ocular samples.

Active trachoma (TF and/or TI) was diagnosed in 23/57 (46%) of all ages, and 22/33 (70%) of 1–9-year-olds. *Ct* was detected in 11/57 (19%) participants of all ages (Table 1), and 8/33 (24%) of 1–9-year-olds. Overall, Ct DNA was detected in 7/22 (32%) of 1-9-year olds with TF and/or TI. All *Ct*-positive samples tested positive for both *omcB* and *pORF2*. Overall, ocular *Ct* was detected in 4/9 (44%) households (“ocular-positive households”).

From these nine households, we collected 202 non-ocular swabs, and detected *Ct* DNA in 17 of them (8.4%) (Table 2A). Positive non-ocular swabs were found in all four ocular-positive households (Table 2B). No positive non-ocular swabs were found in the five ocular-negative households (Table 2C). All five air control swabs were negative. In addition to the non-ocular swabs, 81 flies were collected and tested (Table 2A). *Ct* DNA was detected in 9/81 (11%) flies. From these 81 flies, 71 (88%) were *Musca sorbens*, 2 (2%) were *Musca domestica* and 8 (10%) were not identified. Positive flies came from three ocular-positive households. All positive flies were collected from five ocular-positive individuals.

**Table 2:** Results from non-ocular swabs (n = 202) and flies (n = 81) collected from 57 individuals living in the nine pilot households.

Variable	Total^a^		*C*. *trachomatis* positive^b^		
	n	%	n	%	(95% CI)
**(A) All households**					
Non-ocular swabs tested	202	100	17	8.4	(5.3–13.1)
Non-ocular swab^c^					
Face^d^	33	16.3	5	29.4	(6.7–30.9)
Hand	42	20.8	7	41.2	(2.5–19.0)
Neckline clothing	42	20.8	3	17.6	(8.3–30.6)
Cuff clothing	42	20.8	1	5.9	(4.2–12.3)
Sleeping surface^e^	30	14.9	0	0.0	(0.0–11.4)
Washing jug	9	4.5	1	5.9	(2.0–43.5)
Large water can	9	4.5	0	0.0	(0.0–29.9)
Air	5	2.5	0	0.0	(0.0–43.4)
Flies tested	81	100.0	9	11.1	(6.0–19.8)
**(B) Ocular *C*. *trachomatis* positive-households**					
Non-ocular swabs tested	90	100	17	18.9	(12.1–28.2)
Non-ocular swab^c^					
Face^d^	15	16.7	5	29.4	(15.2–58.3)
Hand	19	21.1	7	41.2	(19.1–59.0)
Neckline clothing	19	21.1	3	17.6	(5.5–37.6)
Cuff clothing	19	21.1	1	5.9	(0.9–24.6)
Sleeping surface^e^	14	15.6	0	0.0	(0.0–21.5)
Washing jug	4	4.4	1	5.9	(4.6–69.9)
Large water can	4	4.4	0	0.0	(0.0–49.0)
Air	3	3.3	0	0.0	(0.0–56.1)
Flies tested	35	100.0	9	25.7	(14.2–42.0)
**(C) Ocular *C*. *trachomatis*-negative households**					
Non-ocular swabs tested	112	100	0	0.0	(0.0–3.3)
Non-ocular swab^c^					
Face^d^	18	16.1	0	0.0	(0.0–17.6)
Hand	23	20.5	0	0.0	(0.0–14.3)
Neckline clothing	23	20.5	0	0.0	(0.0–14.3)
Cuff clothing	23	20.5	0	0.0	(0.0–14.3)
Sleeping surface^e^	16	14.3	0	0.0	(0.0–19.4)
Large water can	5	4.5	0	0.0	(0.0–43.4)
Washing jug	5	4.5	0	0.0	(0.0–43.4)
Air	2	1.8	0	0.0	(0.0–65.8)
Flies tested	46	100.0	0	0.0	(0.0–14.3)

Abbreviations: CI, confidence interval.

^a^Shows column percentage compared to all non-ocular swabs.

^b^Shows row percentage to indicate the proportion of each non-ocular swab that tested positive or negative.

^c^Data missing for 7 children and 8 male adults; 4 children and 4 male adults from positive households and 3 children and 4 male adults from negative households

^d^Face swabs were only collected from children

^e^Sleeping surfaces were only swabbed where children’s faces rest (according to primary caregiver)

### Population-based survey

A total of 1220 individuals were enrolled from 247 households in one village between 11^th^ April and 25^th^ June 2018. The median age was 11 years (range 1–80 years) and 633/1220 (52%) were female (Table 3). Ocular discharge was observed in 337/1220 (28%) participants, 281/1220 (23%) had nasal discharge and 452/1220 (37%) had flies present on their face at the time of examination (Table 3).

**Table 3:** Characteristics 1220 individuals living in 247 households in the population-based survey, by ocular *C*. *trachomatis* qPCR status.

Variable	Total (n = 1220)		*C*. *trachomatis* positive (n = 21)^a^	
	n	%	n	%
Age in years continuous				
Median (IQR)	11	(6–28)	7	(5–9)
Age years categorical				
1–9	533	43.7	17	81.0
10–17	223	18.3	2	9.5
18+	464	38.0	2	9.5
Sex				
Female	633	51.9	9	42.6
Male	587	48.1	12	57.1
Presence of ocular discharge				
Yes	337	27.6	13	61.9
Presence of Nasal discharge				
Yes	281	23.0	11	52.4
Presence of flies on the face				
Yes	452	37.0	12	57.1
Presence of TF				
Yes	112	9.2	13	61.9
Presence of TI				
Yes	21	1.7	5	23.8
Presence of TF and or TI				
Yes	119	9.8	15	71.4
*omcB* load in copies/μL				
Median IQR)	198.6	(23.2–3189.1)	198.6	(23.2–3189.1)
*pORF2* load in copies/μL				
Median (IQR)	120.9	(4.79–702.8)	120.9	(4.79–702.8)

Abbreviations: TF, trachomatous inflammation—follicular; TI trachomatous inflammation—intense; IQR, Inter quartile range.

^a^Shows column percentage compared to all *Chlamydia trachomatis* qPCR positive ocular samples.

### *C*. *trachomatis* detection in ocular samples

**Fig 1:**
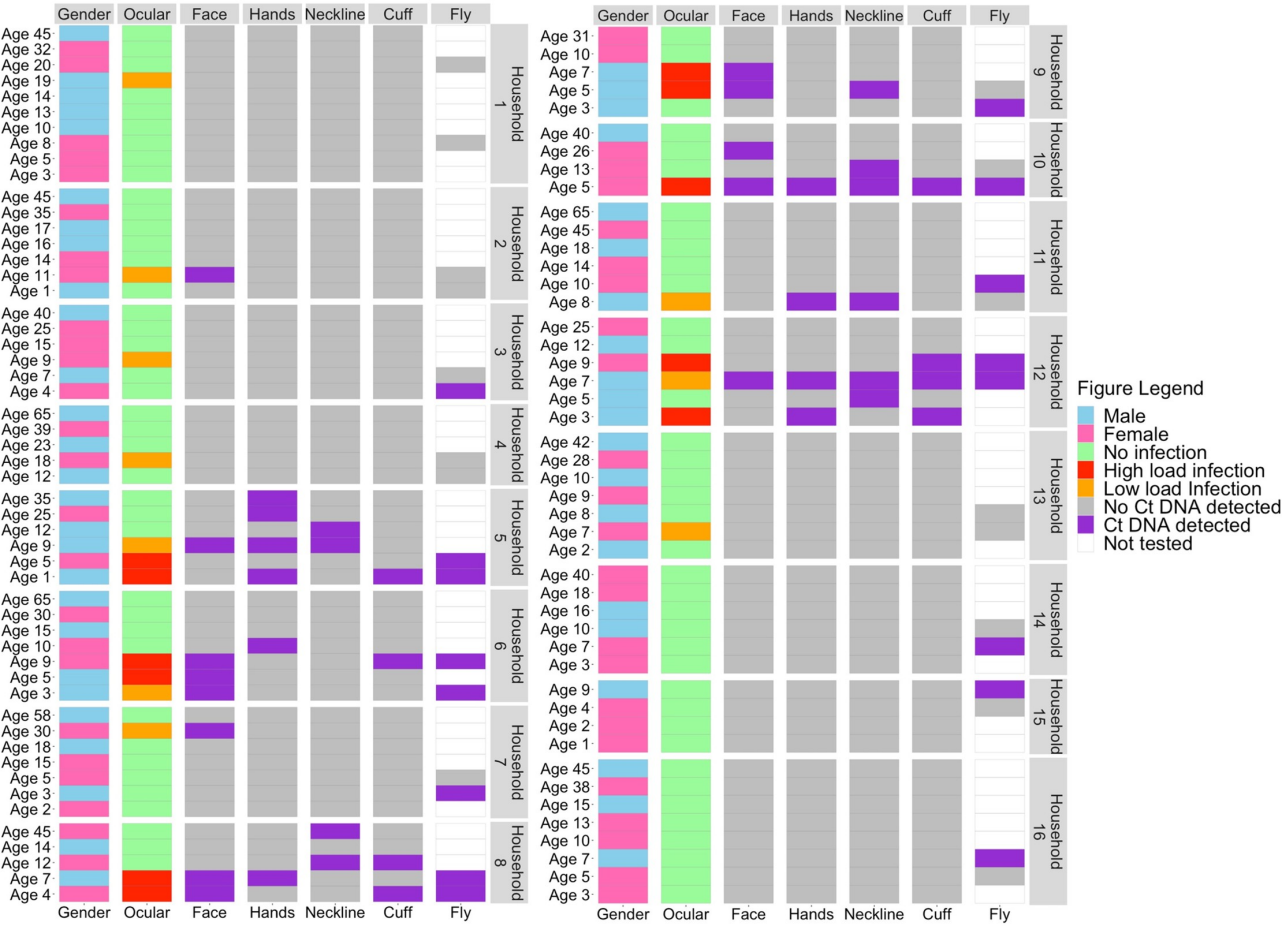
Heatmap showing the presence or absence of *C*. *trachomatis* DNA. Data is shown by age and gender of each household member within 13 ocular-positive households and 3 ocular-negative households. Each divided block represents a separate household. Each row represents one household member with age of that member in front of the row.

Active trachoma (TF and/or TI) was diagnosed in 119/1220 (9.8%) of all ages, and 111/533 (20.8%) of 1–9 year-olds. *Ct* DNA was detected in 21/1220 (1.7%) all-ages participants, including 17/533 (3%) 1–9-year-olds (Table 3; Fig 1). Overall, Ct DNA was detected in 13/111 (12%) 1-9-year olds with TF and/or TI. All *Ct*-positive samples tested positive for both *omcB* and *pORF2*. We detected a median *omcB* load of 198.6 copies/μL among positive samples. Overall, ocular *Ct* was detected in 13/247 (5%) households.

### *C*. *trachomatis* detection in non-ocular samples

We collected 5470 non-ocular swabs and 3977 flies from the 247 households. Informed by the pilot study, we decided not to test all samples collected from ocular-negative households. Instead, in addition to testing swabs and flies from all ocular-positive households, we randomly selected 15 ocular-negative households for sample testing. Comparison of the 13 ocular-positive and 15 ocular-negative households provided no evidence of a difference in terms of age, gender, facial cleanliness or clinical signs amongst household residents (Table 4).

**Table 4:** Comparison of the characteristics of individuals in 13 ocular *C*. *trachomatis*-positive households and 15 randomly selected ocular *C*. *trachomatis* negative households.

Variable	Total (n = 162)	Ocular-negative household (n = 80)		Ocular-positive household (n = 82)			
		n	%	n	%	n	%	P-value
Age in years continuous							
Median (IQR)	10	(5–25)	9	(4–25)	12	(7–28)	0.079
Age years categorical							
1–9	75	46.3	43	53.8	32	39.0	0.091
10–17	35	21.6	11	13.8	21	25.6	
18+	55	34.0	26	32.5	29	35.4	
Sex							
Female	81	50.0	42	52.5	39	47.6	0.530
Male	81	50.0	38	47.5	43	52.4	
Presence of ocular discharge							
Yes	54	33.3	23	28.8	31	37.8	0.222
Presence of Nasal discharge							
Yes	48	29.6	24	30.0	24	29.3	0.918
Presence of flies on the face							
Yes	60	37.0	30	37.5	30	36.6	0.904
Presence of TF							
Yes	37	22.8	21	26.3	16	19.5	0.307
Presence of TI							
Yes	10	6.2	3	3.8	7	8.5	0.637
Presence of TF and/or TI							
Yes	40	24.7	21	26.3	19	23.2	0.650

Abbreviations: TF, trachomatous inflammation—follicular; TI trachomatous inflammation—intense; IQR, Inter quartile range.

In total, 751/5470 (14%) non-ocular swabs were tested; 369 (49%) were from 13 ocular-positive households and 381 (51%) were from the 15 randomly selected ocular-negative households. In total, 43/751 (6%; 95% confidence interval, CI: 4–7%) non-ocular swabs were positive for *Ct* DNA (Table 5A). Positive non-ocular swabs were found in 9/13 ocular positive households (Fig 1). The non-ocular sites that yielded the highest proportion of positive swabs were faces (16%; 95% CI: 10–26%) followed by necklines from clothing (14%; 95% CI: 8–23%), hands (11%; 95% CI: 6–20%), cuffs of clothing (10%; 95% CI: 5–18%), a washing jug (8%; 95% CI: 14–33%) and a sleeping surface (6%; 95% CI: 1–28%) (Table 5B; Fig 1). From these positive non-ocular swabs, 31/43 (72%) were collected from 16 ocular-positive persons, of whom 11 had high-load ocular infections and 5 had low-load ocular infections (Fig 1). Ten positive non-ocular swabs were collected from nine ocular-negative persons living in five high-load ocular-positive households (Fig 1). The remaining two positive swabs were from a sleeping surface and water jug that were not person specific, but both were found in the same high-load ocular-positive household. No positive non-ocular swabs were found in any household without ocular infection (Table 5C). All 13 (1.6%) air control swabs were negative for *Ct* DNA.

**Table 5:** Results from non-ocular swabs (n = 751) and flies (n = 288) collected from 162 individuals living in 28 households.

Characteristic	Total^a^		*C*. *trachomatis* positive^b^		
	n	%	n	%	(95% CI)
**(A) All households**					
Non-ocular swabs tested	751	100.0	43	5.7	(4.3–7.6)
Non-ocular swab					
Face	162	21.6	13	8.0	(4.8–13.2)
Hand	162	21.6	9	5.6	(3.0–10.2)
Neckline clothing	162	21.6	11	6.8	(3.8–11.7)
Cuff clothing	163	21.7	8	4.9	(2.5–9.4)
Sleeping surface	33	4.4	1	3.0	(0.5–15.3)
Washing jug	28	3.7	1	3.6	(0.6–17.7)
Large water can	28	3.7	0	0.0	(0.0–12.1)
Air	13	1.7	0	0.0	(0.0–22.8)
Flies tested	288	100.0	35	12.2	(8.9–16.4)
**(B) Ocular C. trachomatis-positive households**					
Non-ocular swabs tested	369	100.0	43	11.7	(8.8–15.3)
Non-ocular swabs					
Face	80	21.7	13	16.3	(9.7–25.8)
Hand	80	21.7	9	11.3	(6.0–20.0)
Neckline clothing	80	21.7	11	13.8	(7.9–23.0)
Cuff clothing	81	22.0	8	9.9	(5.1–18.3)
Sleeping surface	16	4.3	1	6.3	(1.1–28.3)
Washing jug	13	3.5	1	7.7	(1.4–33.3)
Large water can	13	3.5	0	0.0	(0.0–22.9)
Air	6	1.6	0	0.0	(0.0–39.0)
Flies tested	129	100.0	31	24.0	(17.5–32.1)
**(C) Ocular C. trachomatis-negative households**					
Non-ocular swabs tested	382	100.0	0	0.0	(0.0–1.0)
Non-ocular swab					
Face	82	21.5	0	0.0	(0.0–4.5)
Hand	82	21.5	0	0.0	(0.0–4.5)
Neckline clothing	82	21.5	0	0.0	(0.0–4.5)
Cuff clothing	82	21.5	0	0.0	(0.0–4.5)
Sleeping surface	17	4.5	0	0.0	(0.0–18.4)
Washing jug	15	3.9	0	0.0	(0.0–20.4)
Large water can	15	3.9	0	0.0	(0.0–20.4)
Air	7	1.8	0	0.0	(0.0–35.4)
Flies tested	159	100.0	4	2.6	(1.0–6.3)

^a^Shows column percentage compared to all non-ocular swabs.

^b^Shows row percentage to indicate the proportion of each non-ocular swab that tested positive or negative.

### *C*. *trachomatis* detection in flies

In total, 288 (7%) of the 3997 captured flies were tested by PCR. The 288 had been caught from 58 children (median age 6 years, range 1–18 years); 159 (55%) were from 15 ocular-negative households and 129 (45%) were from 13 ocular-positive households (Table 5). From these 288 flies, 260 (90%) were *Musca sorbens*, 19 (7%) were *Musca domestica* and 9 (3%) were not identified. *Ct* DNA was detected in 35/288 (12%; 95% CI: 9–16%) flies (Table 5A). We were more likely (OR 12.3; 95% CI 4.2–35.8) to find *Ct-*positive flies in ocular-positive households (24%; 95% CI: 18–32%; Table 5B) than in ocular-negative households (3%; 95% CI: 1–6%; Table 5C). Among *Ct*-positive flies, 24/35 (71%) were collected from eight individuals with ocular infection, of whom seven had high-load ocular infections and one had a low-load ocular infection. Seven (18%) *Ct-*positive flies were collected from the faces of five ocular-negative persons, of whom two were living in a two high-load ocular-positive households and three were living in three low-load ocular-positive households. Overall, *Ct-*positive flies were found in 9/13 (69%) ocular-positive households (Fig 1). The remaining four *Ct-*positive flies were collected from three ocular-negative persons living in three ocular-negative households (Fig 1).

### Geospatial distribution of *C*. *trachomatis* infections

A scree plot was generated which displays the proportion of the total variation based on geographical distance between household GPS coordinates that can be explained by each added k-mean cluster based on the total within sum of squares. The scree plot (S1 Fig) shows that the slope of the curve is levelling off after two k-mean clusters indicating little added value from grouping the data in more than two k-mean clusters. Cluster 1 (consisting of 7 households) included predominantly high-load ocular-positive households (71%; 5/7), whereas Cluster 2 (6 households) included predominantly low-load ocular-positive households (83%; 5/6).

**Fig 2:**
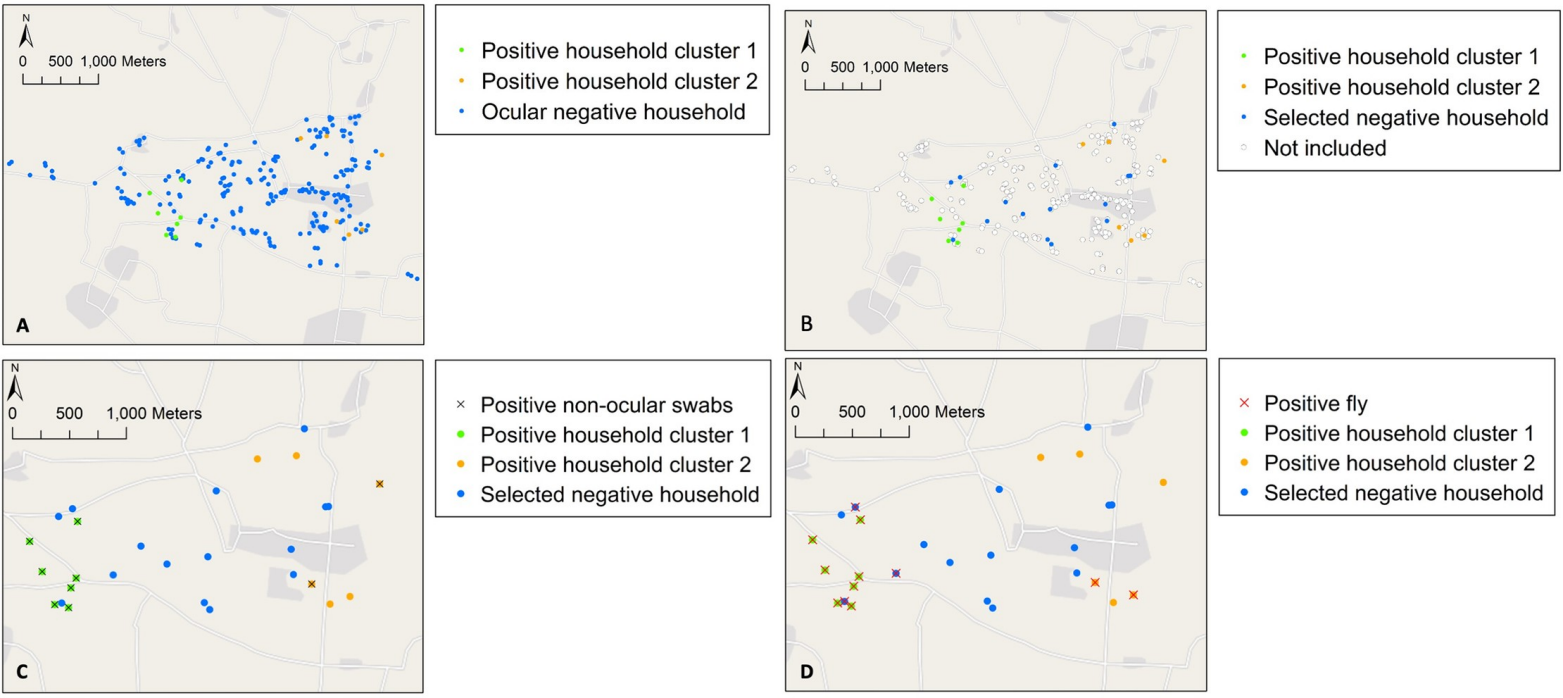
Geographical maps. Showing (A) the geographical distribution of ocular *C*. *trachomatis-*positive and negative households by cluster, (B) the geographical distribution of *C*. *trachomatis-*positive and randomly selected *Ct-*negative households, (C) the geographical distribution of *C*. *trachomatis-*positive non-ocular samples and (D) the geographical distribution of *C*. *trachomatis-*positive flies. Geographical maps were created using ArcGIS 10.5 (ESRI Inc., USA) and OpenStreetMap.

Maps show the distribution of ocular-positive and -negative households (Fig 2A) and the locations of the 15 randomly-selected ocular-negative households (Fig 2B). These maps show that positive non-ocular swabs were predominantly found in Cluster 1 (Fig 2C). Most positive flies were found in Cluster 1 and positive flies found in ocular-negative households were all close to Cluster 1 (Fig 2D).

## Discussion

In this study, we identified biologically plausible trachoma transmission routes by systematically testing flies and non-ocular swabs collected from hands, faces, clothing, sleeping surfaces, and water containers for *Ct* DNA. We detected *Ct* DNA on both non-ocular swabs and flies.

Results were consistent between the pilot study and the main population-based survey. *Ct* was only detected in non-ocular swab samples from ocular-positive households. Moreover, the large majority of positive non-ocular swabs were collected in high-load ocular-positive households. This suggests that transmission is more likely to occur within households in which an ocular-positive person resides, a conclusion also supported by the high degree of clustering seen at household level [23, 24].

In ocular-positive households, *Ct* DNA was most frequently detected on faces, hands and clothing, being found in such locations in 10–16% of samples tested. The presence of *Ct* DNA at non-ocular sites suggests that these sites are plausible routes for transmission. It also indicates that it might be important for trachoma programs to address hand and clothes washing in addition to facial cleanliness.

It is noteworthy that 25% of flies caught in ocular-positive households were *Ct*-positive, compared with 3% in ocular-negative households. This is a substantially higher positivity proportion than for any of the surface swabs sites we tested. The majority of *Ct*-positive flies collected in the population-based survey were caught from children with high-load *Ct* infections or living in high-load *Ct-*positive households. We caught *Ct*-positive flies from the faces of children without ocular infection who were living in ocular-positive households, providing direct evidence that flies could contribute to within-household transmission. Interestingly, *Ct*-positive flies were also caught from children in ocular-negative households in close proximity to ocular-positive households, in a predominantly high-load ocular positive cluster (Fig 2D, Cluster 1). This suggests that flies may also have the potential to contribute to transmission of trachoma both within and between households. Further investigation is critical.

Three previous studies tested wild-caught *M*. *sorbens* for the presence of *Ct* DNA [11, 12, 25]. The first, from The Gambia, reported that 0.5% (2/395) of flies caught from children with active trachoma were *Ct*-positive [25]. The second found *Ct* on 15% (15/103) of flies caught from children in three villages in Ethiopia, where 50% of children had active trachoma; no data on ocular infection in humans was provided [12]. The third reported that 23.0% of flies caught from children in untreated Ethiopian villages carried detectable *Ct*; the children themselves had an ocular *Ct* prevalence of 30%. In comparison, they reported a prevalence of 0.5% in flies caught from children in MDA-treated villages, with an ocular *Ct-*prevalence of 1.2–2.3% [11]. All of the earlier studies used DNA amplification techniques, but none reported the individual-level relationship between human-ocular and fly *Ct* detection.

Potential limitations of our study should be noted. Although we defined the presence of ocular and nasal discharge consistently during the study, intra- and inter-observer assessments were not undertaken and photographs were not taken for this purpose. Thus the measures of facial cleanliness are subjective and a more objective measure is required to fully assess the relationship between active trachoma, *Ct* and facial cleanliness. We used qPCR to detect the presence of *Ct* DNA. DNA detection does not necessarily reflect the presence of viable organisms. Although this does not invalidate the possibility that our *Ct*-positive sites form part of the routes for transmission, further work is needed to determine whether detected *Ct* DNA originates from viable organisms and how long any viable organisms remain viable at non-ocular sites or on flies. We only found 12% of non-ocular swabs from ocular-positive households to be positive. This could potentially be an underestimation of the true prevalence of *Ct* in non-ocular sites, as it is impossible to swab complete surfaces. However, we aimed to standardize sampling as much as possible by collecting each swab in a systematic manner as described above. Finally, generalizability of results to other villages in Ethiopia or other countries may be limited since we only included 2 villages in the pilot study and 1 village in the population-based study. Further research is needed to determine if these results are systematically found in other geographical locations.

In conclusion, we found *Ct* DNA to be present on the hands, faces and clothing of individuals living in households in which one or more residents had detectable ocular *Ct* infections, suggesting that this might be a route of transmission within *Ct* infected households. In addition, we detected *Ct* DNA relatively frequently on flies in ocular-positive households and occasionally in negative households, suggesting that flies might be a vector for transmission within and between *Ct* infected and uninfected households. Overall, these findings suggest there may be several plausible ocular *Ct* transmission routes between people, within and between households, which would need to be simultaneously addressed within communities to suppress transmission.

## Supporting information

S1 Checklist STROBE checklist. (DOCX)

S1 Fig Scree plot based on the geographical distance of positive households (n = 13) showing the total within sum of square for each number of k clusters. The figure shows a clear kink in the curve at 2 clusters after which the curve evens out suggesting that k-means clustering using 2 clusters is optimal.
(TIF)
