# Supplementary figures and images for: Detecting extra-ocular Chlamydia trachomatis in a trachoma-endemic community in Ethiopia: Identifying potential routes of transmission
Source: PLoS Negl Trop Dis. 2020 Mar 4;14(3):e0008120. doi: 10.1371/journal.pntd.0008120 (PMC7075638; doi:10.1371/journal.pntd.0008120)

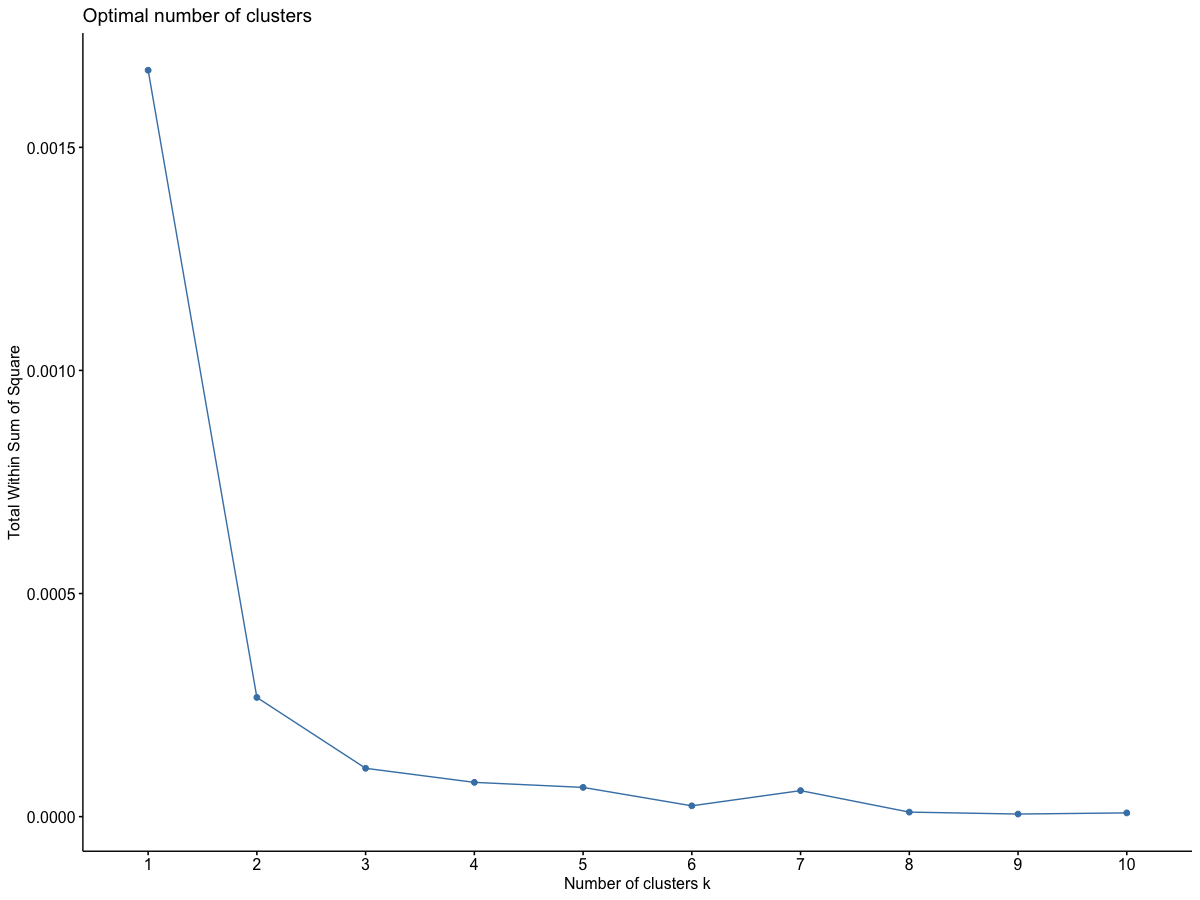

Supplement: S1 Fig — The figure shows a clear kink in the curve at 2 clusters after which the curve evens out suggesting that k-means clustering using 2 clusters is optimal. (TIF) [file pntd.0008120.s002.tif]
